# The Microscopist

**Published:** 1853-11

**Authors:** 


*The Microscopist, by* JOHN H. WYTHES, M. D. Philadelphia: Lindsay & Blakiston.

THIS little work contains directions for using the microscope and also for preparing and mounting objects. It also contains chapters on minute, dissection, injection, &c.

As there are many physicians who are using the instrument, and many others who are desirous of doing so, who feel the embarrassment of having to learn everything from experience, we quote a part of the chapter on mounting and preserving objects for examination,

TRANSPARENT OBJECTS.—Transparent objects are mounted on slips of glass, the size of which, as adopted by the Microscopic Society of London, is 3 inches by 1 inch, or 2 inches by 1½ inches. The French opticians, however, prepare many of their slides 2¼ inches by ⅝ths of an inch, and this size is most frequently imported into the United States; indeed, a larger size is unsuitable for many of the French instruments, although to be preferred on other accounts.

There are three methods of mounting transparent objects. 1st, in the dry way—in which the object is simply placed on the glass, and covered with a thin glass cover, cemented by its edges to the under piece, with sealing-wax, varnish, &c.

2dly. In some preservative fluid.

3dly. In Canada balsam.

The glass slides should be clear, free from veins and bubbles, uniform length and breadth, and should have their edges ground smooth by rubbing them on a flat cast-iron plate with emery and water.

Sections of teeth and bone, and of some kinds of wood, hairs of animals, scales of butterflies, test objects from the infusoria, &c., are best mounted dry; but all very delicate animal and vegetable tissues, to exhibit their structure clearly, should not be mounted in the dry way, nor in Canada balsam, but in some preservative fluid.

Dr. Goadby has devoted much time to this subject, and has succeeded in supplying to the microscopist a ready, cheap, and effectual means for mounting animal structures with the greatest possible ease and security. Dr. G. received a gold medal from the Society of Arts for his invention. He has kindly furnished me with the following description of his preserving fluids:

"A 1. Bay salt (coarse sea-salt), 4 ounces,
Alum, 2 ounces,
Corrosive sublimate, 2 grains,
Boiling water, 1 quart.

"A 2. Bay salt, 4 ounces,
Alum, 2 ounces,
Corrosive sublimate, 4 grains,
Boiling water, 2 quarts.

"The A 1 fluid is too strong for most purposes, and is only to be employed where great astringency is required to give form and support to delicate structures.

"The A 2 fluid may be very extensively used, and is best adapted for permanent preparations; but neither of these fluids should be used in the preservation of animals possessing any carbonate of lime (all the Mollusca), as the alum becomes decomposed, and the sulphate of lime is formed and precipitated and the animal spoiled. For such use the

"*B fluid, specific gravity* 1·100.
Bay salt, 8 ounces,
Corrosive sublimate, 2 grains,
Water, 1 quart.

"Marine animals require a stronger fluid of this kind, viz., specific gravity 1·148, which is made by adding more salt (about 2 ounces) to the above.

"The corrosive sublimate is used to prevent vegetation growing in the fluid, and no greater quantity should be used than 2 grains per quart of fluid; but, as it coagulates albumen, it must be left out when ova are to be preserved, or when it is desired to maintain the transparency of certain tissues."

Mounting in Fluid.—The most minute structures, such as the vessels of plants, and the muscular and other tissues of animals, requiring in all cases high powers for their proper exhibition, must of necessity be preserved in very thin cells with a small amount of fluid.

On a slip of glass, 3 inches by 1, cleaned by a solution of caustic potash to remove all grease, lay a drop of the fluid; put the object in this and spread it out with the point of a needle, &c.—Select a thin and flat glass cover, clear it likewise from grease, &c., touch its edges with cement, and drop it gently over the object.—Press it lightly to exclude the excess of fluid, which can be re-removed by strips of blotting-paper. Then cement the edges of the cover to the bottom glass. Care must be taken to exclude all air-bubbles from between the glasses. Objects mounted thus do not keep long, and it is best to make a thicker cell. This may be made by painting a round or square ring on the slip with some sort of cement which will not be acted upon by the fluid employed.—White lead worked with 1 part linseed oil and 3 of spirits of turpentine is well adapted for this purpose.—In this ring, the fluid and object are placed and the cover put on.

Mounting in Balsam.—Before objects are mounted in Canada balsam they should be perfectly clear and free from moisture.—They are commonly soaked in turpentine, especially opaque objects, as it renders them more transparent. Grease may be removed by sulphuric ether.

Very thin and transparent objects become indistinct in balsam; they should be made dark. Vegetable matters may be charred between two plates of glass over a lamp. Other structures which cannot be charred, may be dyed by soaking in a decoction of fustic or logwood, or a weak tincture of iodine.

The balsam should be warmed on the slide to expel the air.—When objects of a cellular nature have to be mounted, if they are such as heat will not much injure, they may be boiled in the balsam; otherwise numbers of air-bubbles will be left in the cells, and the true structure cannot then be made out satisfactorily.—The extra degree of heat will expand the air and cause it to escape, and the balsam will take its place.

Some object of a tubular nature, such as the trachæ of insects, are better seen if air be contained in the tubes; they will then exhibit the spiral fibre in their interior; but a tracheal tube filled with balsam does not show the fibre at all, the balsam having made all the parts transparent. Small insects, such as fleas, and the parasites of animals, when not overheated, show the ramifications of the trachæ, but those which have been soaked long in turpentine, or have had the air expelled by heat, do not exhibit the spiral markings except under polarized light.

When air is to be got rid of, the heat must be high; otherwise, the use of turpentine must be avoided, the heat of the balsam kept low, and the mounting accomplished quickly.

The best way to heat the balsam on the slide is to place the slide on a small table made of iron or tin, to which a spirit-lamp is applied, as first suggested by Dr. Goadby; yet with careful management a spirit-lamp will do alone.

These extracts show the practical character of the work. It is not designed for the experienced Microscopist, but for those just entering upon this interesting department of study. To such the little volume before us, will be valuable, and we take pleasure in recommending it for their perusal.

It may be had of D. B. Cooke & Co., 135 Lake st. Chicago. J.

